# Tuberous Sclerosis Complex: A Roadmap for Future Research

**DOI:** 10.15844/pedneurbriefs-30-7-1

**Published:** 2016-07

**Authors:** Anna Jeong

**Affiliations:** 1 Department of Neurology, Washington University School of Medicine, St. Louis, MO

**Keywords:** Tuberous Sclerosis Complex, Epilepsy, Autism, mTOR

## Abstract

Investigators from the NINDS and the Tuberous Sclerosis Alliance sponsored a workshop in March 2015, which joined basic scientists and clinicians with expertise in various aspects of Tuberous Sclerosis Complex (TSC), in order to assess the current state of TSC research and to set future goals.

Investigators from the NINDS and the Tuberous Sclerosis Alliance sponsored a workshop in March 2015, which joined basic scientists and clinicians with expertise in various aspects of Tuberous Sclerosis Complex (TSC), in order to assess the current state of TSC research and to set future goals. Research priorities included characterizing the widely variable clinical phenotype associated with TSC, gaining a better understanding of the TSC signaling pathway/network, and improving TSC models. Additional goals include the development of clinical biomarkers to assist in assessing disease burden, prognosis, and potential response to treatment. Lastly, treatment gaps remain for the symptoms associated with TSC, highlighting the need for translation to therapies and the execution of clinical trials. [1]

COMMENTARY. Since the initial clinical description by Désiré-Magloire Bourneville in the late 19th century, extraordinary progress has been made in the understanding and treatment of TSC. Gene discovery and the elucidation of the mTOR signaling pathway have led to targeted drug therapy using rapamycin and rapamycin analogs. These compounds have been tested in randomized, placebo-controlled, double-blind studies [2, 3] and have become standard of care in the medical treatment of the tumors associated with TSC.

TSC is a unique disease that is poised to augment our understanding of more commonly encountered neurological conditions, for example epilepsy and autism. Epilepsy affects over 85% of individuals with TSC, with refractory epilepsy being seen in approximately one-third of those affected and refractory epilepsy, early age of seizure onset, and infantile spasms being associated with poor cognitive outcomes [4]. Additionally, individuals with TSC are at high risk for TSC-associated neurocognitive deficits (TAND), including autism spectrum disorder (ASD), intellectual disability, and mood disorders. TSC is one of the most common single-gene disorders associated with ASD [5]. TSC has emerged as a model disease for study, with one of the most exciting areas being the approach to and treatment of neurological symptoms such as epilepsy and ASD. TSC is a unique disease, in that individuals may be diagnosed early due to a positive family history or systemic disease manifestations, prior to the development of epilepsy or signs of ASD. This early detection may allow for a potential treatment window, with the ultimate goal being truly disease-modifying, preventative treatment. A recent prospective study examining EEG biomarkers in infants found that EEG abnormalities demonstrated 100% positive predictive value in the development of epilepsy [6]. This serves as a first step in the identification of candidate patients, who may benefit from early, possibly pre-symptomatic treatment.

The workshop lays out a roadmap for future research, by outlining the current state of the field and by looking forward to the preclinical and clinical aims that must be met in order to better understand and treat TSC.
